# Root and bone changes in teeth undergoing pre-prosthetic orthodontics before endodontic retreatment prior to implant surgery: 3D evaluation

**DOI:** 10.6026/973206300222905

**Published:** 2026-05-31

**Authors:** Robin Malik, Kalyani Y Joshi, Rakesh Kumar Verma, Manjiri Salkar, Ashtha Arya, Maheswari Eluru

**Affiliations:** 1 Department of Orthodontics & Dentofacial Orthopedics, Santosh Dental College, Santosh (Deemed to be) University, Ghaziabad, NCR Delhi, India; 2 Department of Dentistry, Mahatma Gandhi Institute of Medical Sciences, Sewagram, Wardha, Maharashtra, India; 3 Department of Periodontics, Maharaja Ganga Singh Dental College & Research Centre, Sriganganagar, Rajasthan, India; 4 Department of Dentistry, MGV's KBH Dental college and hospital, Nashik, Maharashtra, India; 5 Department of Conservative Dentistry and Endodontics, SGT Dental College, Hospital and Research Institute, SGT University, Gurugram, Haryana, India; 6 Department of Biomedical Informatics, College of Health Solutions, Arizona State University, USA

**Keywords:** Pre-prosthetic orthodontics, endodontic retreatment, dental implants, root resorption, alveolar bone remodeling, cone beam computer tomography (CBCT), cortical bone thickness, bone density

## Abstract

Loss of alveolar bone and risk of root resorption during pre-prosthetic orthodontic treatment can compromise successful implant placement and long-term stability. Therefore, it is of interest to evaluate three-dimensional changes in root morphology and alveolar bone in teeth undergoing pre-prosthetic orthodontic treatment combined with endodontic retreatment prior to implant placement. Hence, a total of 40 teeth from adult patients were assessed using pre- and post-treatment CBCT scans to measure root length, cortical bone thickness, alveolar crest height, and bone density. The results showed a mild but significant reduction in root length, indicating minimal external root resorption. Significant increases in buccal and lingual cortical bone thickness and bone density were observed, while alveolar crest height remained stable. This interdisciplinary approach appears safe and effective, providing favorable bone conditions for predictable implant placement and long-term success.

## Background:

Dental implant therapy is a predictable and widely accepted option for replacing missing teeth. Its success depends on adequate pre-surgical preparation of hard and soft tissues. Interdisciplinary management involving orthodontics and endodontics is often required before implant placement to optimize site conditions [1]. Pre-prosthetic orthodontics helps correct tooth position, redistribute space and improve occlusion. It also enhances alveolar bone morphology, facilitating implant placement and prosthetic outcomes [2]. Orthodontic tooth movement induces biological remodeling in the periodontal ligament and alveolar bone. Controlled forces cause bone resorption on the pressure side and bone formation on the tension side [3]. These changes result in alterations in root position, morphology and bone dimensions. Although beneficial, excessive orthodontic forces may lead to root resorption, cortical bone thinning, or alveolar defects [4]. Such changes are critical in implant cases as they affect bone volume and implant stability. Endodontic retreatment is essential for teeth with persistent periapical pathology prior to implant planning. Infected root canals may compromise periapical bone and affect adjacent implant sites [5]. Successful retreatment promotes healing and preserves alveolar bone integrity [6]. The combined effects of orthodontic movement and endodontic healing may lead to dynamic changes in root and bone structure. These changes require careful monitoring during treatment. Cone-beam computed tomography (CBCT) has improved the evaluation of three-dimensional root and bone changes. It provides accurate visualization of root length, angulation, cortical thickness and bone quality [7]. CBCT is especially useful in detecting subtle root resorption and alveolar bone defects. It also helps assess changes in alveolar crest height not visible in conventional radiographs [8]. In implant planning; 3D evaluation allows accurate assessment of bone availability and quality. It also aids in determining extraction timing and implant positioning for optimal outcomes [9]. Therefore, it is of interest to evaluate three-dimensional changes in root morphology and alveolar bone in teeth undergoing pre-prosthetic orthodontic treatment combined with endodontic retreatment prior to implant placement.

## Methodology:

This original retrospective observational study was conducted to evaluate three-dimensional changes in root morphology and alveolar bone in teeth undergoing pre-prosthetic orthodontic treatment and endodontic retreatment prior to implant surgery. Ethical approval was obtained from the institutional review board before data collection. A total sample of 40 teeth from 40 patients aged 18 years and above was included based on availability of complete clinical records and diagnostic-quality CBCT scans. The sample size was considered adequate to detect clinically meaningful differences between pre-treatment and post-treatment measurements with sufficient statistical power. Patients selected had undergone orthodontic space creation or alignment followed by endodontic retreatment in the same region prior to planned implant placement. Only cases with both pre-treatment and post-treatment CBCT scans were included. Patients with systemic conditions affecting bone metabolism, history of trauma to the region of interest, advanced periodontal disease, or incomplete records were excluded. All CBCT scans were obtained using the same imaging unit with standardized exposure parameters. Pre-treatment CBCT scans were acquired before the initiation of orthodontic or endodontic procedures, while post-treatment scans were taken after completion of orthodontic movement and endodontic healing but before implant surgery. Three-dimensional measurements were performed using dedicated CBCT analysis software. Root length was measured from the cemento-enamel junction to the root apex along the long axis of the tooth, while alveolar bone assessment included buccal and lingual cortical bone thickness, alveolar crest height and bone density measured at standardized reference points. All measurements were performed by a single calibrated examiner to ensure consistency. Statistical analysis was carried out using appropriate software to compare pre-treatment and post-treatment values and a p-value of less than 0.05 was considered statistically significant.

## Results:

A total of 40 teeth from 40 patients were included in the final analysis. All selected cases had complete pre-treatment and post- treatment CBCT records suitable for three-dimensional evaluation. Descriptive statistics for root and alveolar bone parameters before and after pre-prosthetic orthodontic treatment and endodontic retreatment are presented in Table 1. Comparison of root length measurements revealed a small but measurable reduction following treatment. The mean root length decreased from 14.26 ± 1.12 mm at baseline to 13.91 ± 1.15 mm after completion of orthodontic movement and endodontic retreatment. This reduction was statistically significant (p < 0.05), indicating mild external root resorption associated with treatment (Table 1). Evaluation of alveolar bone changes demonstrated a significant increase in buccal and lingual cortical bone thickness following treatment. The mean buccal cortical bone thickness increased from 1.12 ± 0.26 mm pre-treatment to 1.38 ± 0.29 mm post-treatment, while lingual cortical thickness increased from 1.24 ± 0.31 mm to 1.51 ± 0.34 mm. Both changes were statistically significant (p < 0.01), suggesting favorable bone remodeling in response to orthodontic space preparation (Table 2). Assessment of alveolar crest height showed no statistically significant difference between pre-treatment and post-treatment values, indicating preservation of marginal bone levels during the treatment phase. In contrast, bone density measurements showed a significant increase post-treatment, reflecting improved bone quality prior to implant surgery. Mean bone density values increased from 612.4 ± 85.6 HU to 689.7 ± 92.3 HU (p < 0.01), as summarized in Table 3. Overall, the results indicate that while minor root length reduction was observed, pre-prosthetic orthodontic treatment combined with endodontic retreatment resulted in favorable alveolar bone changes without compromising marginal bone height, thereby creating a suitable foundation for subsequent implant placement.

## Discussion:

The present study evaluated the three-dimensional changes in root morphology and alveolar bone in teeth undergoing pre-prosthetic orthodontic treatment combined with endodontic retreatment prior to implant surgery. The results demonstrate that while mild root shortening occurs, orthodontic treatment along with endodontic management leads to favorable alveolar bone remodeling, emphasizing the importance of interdisciplinary planning in implant site preparation. Mild root resorption observed in this study is consistent with previously reported orthodontically induced external apical root resorption (EARR), which occurs as a biological response to continuous orthodontic forces. External root resorption is influenced by multiple factors, including the magnitude and duration of force, type of tooth movement and patient-specific susceptibility. The minimal resorption noted here suggests that controlled orthodontic forces can achieve desired tooth movement without compromising root integrity [10, 11]. The study showed a significant increase in buccal and lingual cortical bone thickness following orthodontic space creation. This finding indicates active bone remodeling in response to orthodontic forces, where tension on the periodontal ligament stimulates osteoblastic activity and bone apposition, while pressure on the opposite side leads to localized resorption. Such remodeling improves alveolar ridge dimensions and enhances bone availability for future implant placement. These observations are in agreement with previous studies demonstrating that orthodontic movement can be strategically used to improve alveolar ridge morphology and reduce the need for invasive grafting procedures [12, 13]. The findings of the present study are in agreement with the studies by White and Alqahtani *et al.,* who reported that orthodontic tooth movement, may cause mild external root resorption, although the changes are usually clinically insignificant when controlled forces are applied. Similar to their observations, the present study demonstrated minimal reduction in root length with stable alveolar bone support following treatment. Alqahtani *et al.* also emphasized the value of three-dimensional CBCT evaluation for accurate assessment of root and bone changes, supporting its usefulness in interdisciplinary treatment planning before implant placement [14, 15]. Bone density also increased significantly following treatment, which may be attributed to the healing of periapical lesions after endodontic retreatment, along with alveolar bone remodeling from orthodontic forces. Increased bone density is clinically important as it correlates with improved primary implant stability and osseointegration. This reinforces the concept that resolving periapical pathology prior to implant placement not only preserves alveolar bone but may enhance bone quality at the site [16, 17]. The use of cone-beam computed tomography (CBCT) was instrumental in detecting subtle changes in root morphology and alveolar bone that may not be apparent on conventional two-dimensional radiographs. CBCT allows for accurate visualization of root length, angulation, cortical thickness, alveolar crest height and bone density, which cannot be reliably obtained from conventional radiographs. The high-resolution imaging capability of CBCT facilitates early detection of subtle root resorption, cortical thinning, or alveolar defects, aiding clinicians in decision-making and optimizing implant site preparation [17, 18]. The findings of this study have significant clinical implications. Interdisciplinary management combining orthodontic space creation with endodontic retreatment ensures that teeth are properly aligned, periapical health is restored and alveolar bone is optimized for implant placement. Careful control of orthodontic force magnitude and duration is essential to minimize root resorption while achieving sufficient alveolar ridge improvement. This strategy allows predictable implant placement, minimizing the need for extensive bone augmentation and reducing surgical complexity. Limitations of the study include its retrospective design, relatively small sample size and the lack of long-term follow-up to evaluate post-implant outcomes. While CBCT provides precise measurements, longitudinal studies assessing the stability of these changes and the long-term survival of implants in sites prepared with this interdisciplinary approach are recommended. Future prospective studies with larger sample sizes and standardized force protocols will help validate these findings. In summary, the present study demonstrates that pre-prosthetic orthodontic treatment combined with endodontic retreatment leads to minor root shortening but favorable alveolar bone remodeling, including increased cortical thickness and bone density, while preserving alveolar crest height. This emphasizes the importance of three-dimensional imaging and careful treatment planning in achieving optimal outcomes for implant surgery. Further supporting the present findings, Nienkemper *et al.* [19] reported that orthodontic tooth movement can induce measurable changes in root morphology and surrounding alveolar bone, highlighting the importance of careful force control to minimize adverse effects such as root resorption. The study emphasized the role of three-dimensional imaging in accurately monitoring these structural changes during treatment. Similarly, recent evidence from Bhuva *et al.*(2025) [20] indicates that interdisciplinary approaches integrating orthodontic and restorative planning significantly improve implant site preparation and long-term clinical outcomes. The authors highlighted that precise assessment of bone quality and morphology is essential for achieving predictable implant success.

## Conclusion:

Pre-prosthetic orthodontic treatment combined with endodontic retreatment results in minimal root resorption and favorable alveolar bone remodeling. The observed increase in cortical bone thickness and bone density, along with preservation of alveolar crest height, indicates that this interdisciplinary approach effectively prepares implant sites without compromising surrounding dental structures. Thus, careful case selection, controlled orthodontic forces and appropriate imaging assessment are essential to achieve predictable outcomes prior to implant surgery.

## Advancement to knowledge:

This study advances current knowledge by providing three-dimensional evidence on root and alveolar bone changes following combined pre-prosthetic orthodontic treatment and endodontic retreatment prior to implant placement. It demonstrates that this interdisciplinary approach results in minimal root resorption while enhancing cortical bone thickness and density, thereby improving implant site conditions and supporting predictable treatment outcomes.

## Figures and Tables

**Table 1:** Comparison of root length before and after treatment

**Parameter**	**Pre-treatment (Mean ± SD)**	**Post-treatment (Mean ± SD)**	**p-value**
Root length (mm)	14.26 ± 1.12	13.91 ± 1.15	< 0.05

**Table 2:** Changes in buccal and lingual cortical bone thickness

**Parameter**	**Pre-treatment (Mean ± SD)**	**Post-treatment (Mean ± SD)**	**p-value**
Buccal cortical thickness (mm)	1.12 ± 0.26	1.38 ± 0.29	< 0.01
Lingual cortical thickness (mm)	1.24 ± 0.31	1.51 ± 0.34	< 0.01

**Table 3:** Changes in alveolar crest height and bone density

**Parameter**	**Pre-treatment (Mean ± SD)**	**Post-treatment (Mean ± SD)**	**p-value**
Alveolar crest height (mm)	2.18 ± 0.42		> 0.05
Bone density (HU)	612.4 ± 85.6	689.7 ± 92.3	< 0.01
